# Association Between Rate of Hypernatremia Correction and Mortality: A Retrospective Cohort Study Across a Regional Health System

**DOI:** 10.7759/cureus.82558

**Published:** 2025-04-19

**Authors:** Hyun S Lee, Keerthi Renjith, Afrah Misbah, Omer Ahmed, Sanjana Ramakrishnan, Mohammad Jawish

**Affiliations:** 1 Department of Medicine, Rochester Regional Health, Rochester, USA

**Keywords:** hypernatremia, hyponatremia, osmotic demyelination syndrome (ods), rapid correction of hypernatremia, rate of correction

## Abstract

Background: Rate of correction in severe hypernatremia remains controversial. Although data increasingly supports rapid correction, hypernatremia is still often treated similarly to hyponatremia with a maximum rate of correction of 8-12 mmol/L per day due to concerns of neurological complications. This retrospective cohort study investigated the association between the rate of correction in hypernatremia and mortality. A secondary objective was to evaluate whether any adverse neurological outcomes were attributable to rapid correction.

Methods: A retrospective cohort study of patients with severe hypernatremia (serum sodium ≥155 mmol/L) was conducted across a health system in the United States between January and December 2023. Rates of correction were calculated using the time between peak serum sodium values and first eunatremic (serum sodium ≤145 mmol/L) or last known values. Patients were categorized by their hypernatremia correction rates into slow (≤8 mmol/L/day) or rapid (>8 mmol/L/day) correction groups. Mortality was compared between the two groups using Fisher’s exact test and survival analysis for 90-day and one-year intervals. Multivariate Cox regression analysis was performed to evaluate for association between the rate of correction and mortality.

Results: Among 150 included patients, 33 underwent rapid correction. The slow correction group had higher Charlson Comorbidity Indices compared to the rapid correction group. No significant differences in 90-day (43% vs 33%, p=0.42) and one-year mortality rates (63% vs 52%, p=0.23) were observed between the slow and rapid correction groups. Subsequent chart review revealed no documented adverse neurological outcomes attributable to rapid correction. Multivariate analysis did not identify a significant association between correction rate and mortality (hazard ratio 1.00, p=0.27).

Conclusion: These findings add to the growing evidence challenging traditional concerns about rapid correction of hypernatremia in adults, suggesting that rapid correction rates exceeding 8 mmol/L/day do not increase mortality or cause adverse neurological events. These results support reconsidering rigid correction limits and highlight the need for further research on individualized treatment strategies.

## Introduction

Hyponatremia and hypernatremia, two common disorders of body tonicity seen in hospitalized patients are defined as the presence of serum sodium concentration outside the normal range of 135-145 mmol/L [1]. Current practice guidelines suggest limiting the rate of correction to no more than 8-12 mmol/L/day in hyponatremia due to concerns of osmotic demyelination syndrome (ODS) at higher rates [2]. The lower correction rate is often recommended for higher-risk groups, including the elderly, chronic alcohol users and malnourished individuals [2]. Although rare, there is evidence to support this approach, with observed cases of ODS in rapid correction of hyponatremia [3]. However, this maximum correction rate has been extrapolated for use in hypernatremia, despite limited evidence of comparable risks in hypernatremia [4]. Furthermore, it is often difficult and resource-intensive to achieve this ideal rate of correction, with patients often over- or under-correcting despite best efforts [5].

There is increasing evidence that a more liberal rate of correction does not cause adverse effects in hypo- or hypernatremia [6-9]. Two studies did not find any association with rapid correction of hypernatremia and adverse outcomes [8,9]. However, since both studies were conducted in quaternary centers, it is unclear whether the results can be generalized to smaller community health systems with different resources and patient outcomes [10].

The aim of this study was to evaluate the association between hypernatremia correction rates and mortality in a community hospital system. A secondary objective of this study was to assess whether any adverse neurological outcomes were seen in patients with rapid correction of hypernatremia.

## Materials and methods

Study design, setting, and patients

This retrospective cohort study was conducted at Rochester Regional Health, Rochester, United States, with approval from the Rochester Regional Health Institutional Review Board (IRB). The health system consists of a 528-bed tertiary hospital, eight community hospitals, five skilled nursing facilities, and multiple outpatient practices [11]. Patients across these centers with severe hypernatremia (defined as serum sodium ≥155 mmol/L) between January 2023 and December 2023 were included in our study. Patients with laboratory values but no accompanying clinical care within the system were excluded. We also excluded patients with no subsequent sodium measurements following the peak serum sodium level. For patients with multiple episodes of severe hypernatremia, the episode with the highest peak serum sodium value was used for analysis. 

Correction rate definition and categories

For each patient, we calculated the overall rate of correction between the peak serum sodium value and the first documented episode of eunatremia. This was calculated using the timestamp of the peak sodium and the first eunatremic values according to the following formula:

$\text{Rate of Correction (mmol/L/day)} = \frac{\text{Peak serum sodium value} - \text{First eunatremic value}}{\text{Time of peak serum sodium} - \text{Time of first eunatremia}}$

For patients who did not reach eunatremia, we used the last known sodium value to calculate the overall rate of correction. The maximal rate of correction was also calculated for each patient, defined as the highest rate of correction between two consecutive sodium levels.

Various cut-offs were used in previous studies to define rapid correction, ranging from 6-12 mmol/L/day [6]. In our study, rapid correction was defined as being greater than or equal to 8 mmol/L/day to increase sensitivity for any adverse events from overcorrection. Sensitivity analysis using the previously studied cut-off value of 12 mmol/L/day was also performed. Identical cut-off values were used to categorize maximal rates of correction into slow and rapid correction groups.

Data collection

Data was extracted from the health system’s shared electronic medical records system (Epic Systems Corporation, Verona, Wisconsin, United States). After identifying patients, we collected the date and time of serum sodium values from the first instance of severe hypernatremia until the first instance of eunatremia (serum sodium ≤145 mmol/L). Dates of death, if available, were obtained, and time to death was calculated from the date of the peak serum sodium value.

Patients with rapid rates of correction underwent further review for possible erroneous lab values and adverse events related to rapid correction. Results were deemed erroneous if there was documentation of a repeat test with corrected results, or if the remainder of the test panel results were indicative of dilutional effect due to intravenous therapies on review by two independent physicians.

Adverse neurological events were defined as any documentation of unexplained worsening mental status, seizures, or, when available, imaging findings of cerebral edema. A manual review of the medical records was performed by two independent physicians to evaluate for the above, with a third physician serving as a tiebreaker for any conflicts.

Additional data regarding patient demographics (age, gender, race/ethnicity), admission and discharge code statuses, comorbidities included in the Charlson comorbidity index (CCI) [12], diuretic (loop, thiazide) use, angiotensin-converting enzyme inhibitor (ACE-i) or angiotensin receptor blocker (ARB) use, sodium-glucose cotransport 2 (SGLT2) inhibitor use and mineralocorticoid receptor antagonist (MRA) use were collected. Information on whether the initial lab value was from an outpatient setting was also collected.

Statistical analysis

Patients with rapid correction rates were compared with patients with slow correction rates. Categorical values were compared using Fisher’s exact test, and the Wilcoxon rank sum test for continuous variables. Deceased patients without documented dates of death were assumed deceased at one year for mortality comparisons. Sensitivity analysis was conducted using alternative assumed dates of death at 30 and 90 days. These patients were excluded from survival analysis. Kaplan-Meier curves were performed to compare mortality at 90 and 365 days. Multivariate analysis for 90-day mortality was performed on patients with known dates of death using Cox proportional hazards regression, with age, sex, CCI scores as a continuous variable, code status on admission, peak sodium levels, and maximum rate of correction as covariates. All analyses were conducted in R version 4.4.2 (R Foundation for Statistical Computing, Vienna, Austria, https://www.R-project.org/).

## Results

A total of 186 patients were identified with severe hypernatremia in our health system in 2023. A total of 150 patients were included in the final analysis after excluding patients under 18 years of age (n=2), lab values without accompanying clinical care (n=24), and no subsequent serum sodium values after the peak level (n=10). Of note, 15 deceased patients did not have a documented date of death. The median age was 72, with a median CCI score of 5, with an interquartile range (IQR) of 3-7. Most patients had severe hypernatremia diagnosed during their inpatient encounter, with three having severe hypernatremia found in the outpatient setting, and three from a nursing home setting.

A total of 33 patients had rapid overall rates of correction (Table 1). Overall median rate of hypernatremia correction was 5.2 mmol/L/day (IQR 3.1-7.6). The median rate of correction in the slow correction group was 4.3 mmol/L/day (IQR 2.7-6.4). The median rate of correction in the rapid correction group was 12.4 mmol/L/day (IQR 9.6-20.2). Compared to the slow correction group, the rapid correction group had a higher proportion of female patients (67% vs 48%; p=0.01), fewer comorbidities (median CCI 4 vs 6, p=0.02), and no patients who were on more than two out of four medications of interest (0% vs 10%, p=0.03). The rapid correction group had a significantly lower proportion of patients with congestive heart failure (CHF) (9% vs 28%, p=0.02).

**Table 1:** Comparison of characteristics and outcomes of adults with hypernatremia by rates of correction IQR: Interquartile Range; CCI: Charlson Comorbidity Index; DNR: Do Not Resuscitate

Parameters	Total (n=150)	Rate of correction ≤ 8mmol/L/day (n= 117)	Rate of correction > 8mmol/L/day (n=33)	p-value
Age, median (IQR)	72 (61-79)	73 (62-80)	69 (58-78)	0.27
Sex, n (%)				
Female	70 (47)	48 (41)	22 (67)	0.01
Male	80 (53)	69 (59)	11 (33)	
CCI Score				
0-3	44 (29)	33 (28)	11 (33)	0.01
4-5	39 (26)	25 (21)	14 (42)	
>5	67 (45)	59 (50)	8 (24)	
Congestive heart failure, n (%)	36 (24)	33 (28)	3 (9)	0.02
Chronic kidney disease, n (%)	29 (19)	26 (22)	3 (9)	0.09
Ethnicity, n (%)				
White	92 (61)	66 (56)	26 (79)	0.07
Black	36 (24)	32 (27)	4 (12)	
Other	22 (15)	19 (16)	3 (9)	
Repeat episodes, n (%)	17 (11)	13 (11)	4 (12)	1.0
Admission code status, n (%)				
DNR	32 (21)	25 (21)	7 (21)	1.0
Full Code	118 (79)	92 (79)	26 (79)	
Discharge code status, n (%)				
Comfort Care	59 (39)	47 (40)	12 (36)	0.34
DNR	29 (19)	25 (21)	4 (12)	
Full Code	62 (41)	45 (38)	17 (52)	
Code status change, n (%)	73 (49)	60 (51)	13 (39)	0.24
Medications, n (%)				
1 medication	35 (23%)	24 (21%)	11 (33%)	0.03
2 medications	24 (16%)	23 (20%)	1 (3%)	
3 medications	8 (5%)	8 (7%)	0 (0%)	
4 medications	3 (2%)	3 (3%)	0 (0%)	
Peak sodium value, median (IQR)	157 (155,160)	157 (155,160)	159 (155,161)	0.29
Mortality, n (%)				
7 days	20 (13)	16 (14)	4 (12)	1.00
30 days	50 (33)	40 (34)	10 (30)	0.83
90 days	61 (41)	50 (43)	11 (33)	0.42
1 year	91 (61)	74 (63)	17 (52)	0.23

Sodium correction rate and mortality

**Figure 1:**
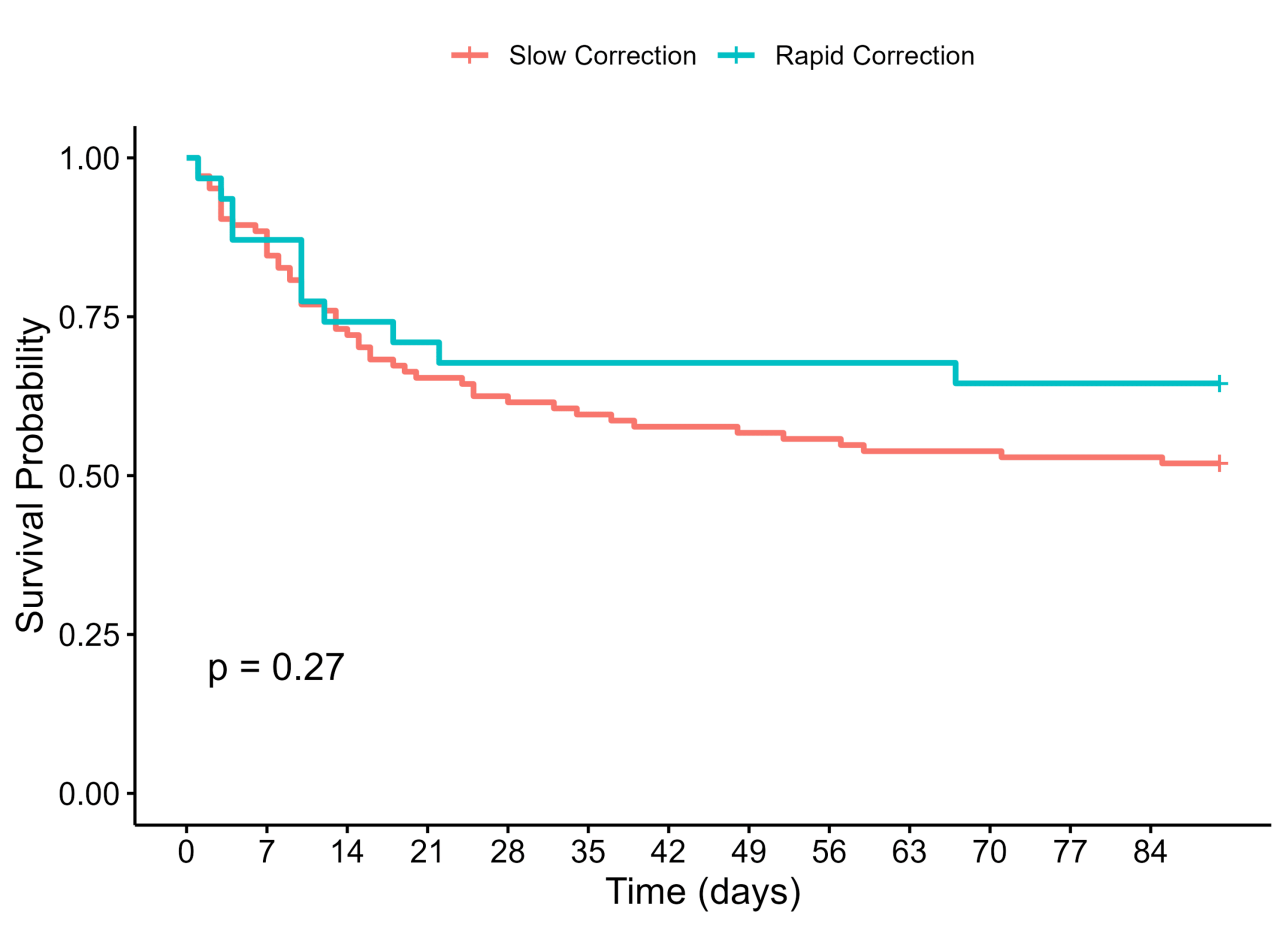
90-day survival curves for rapid versus slow correction groups

**Figure 2:**
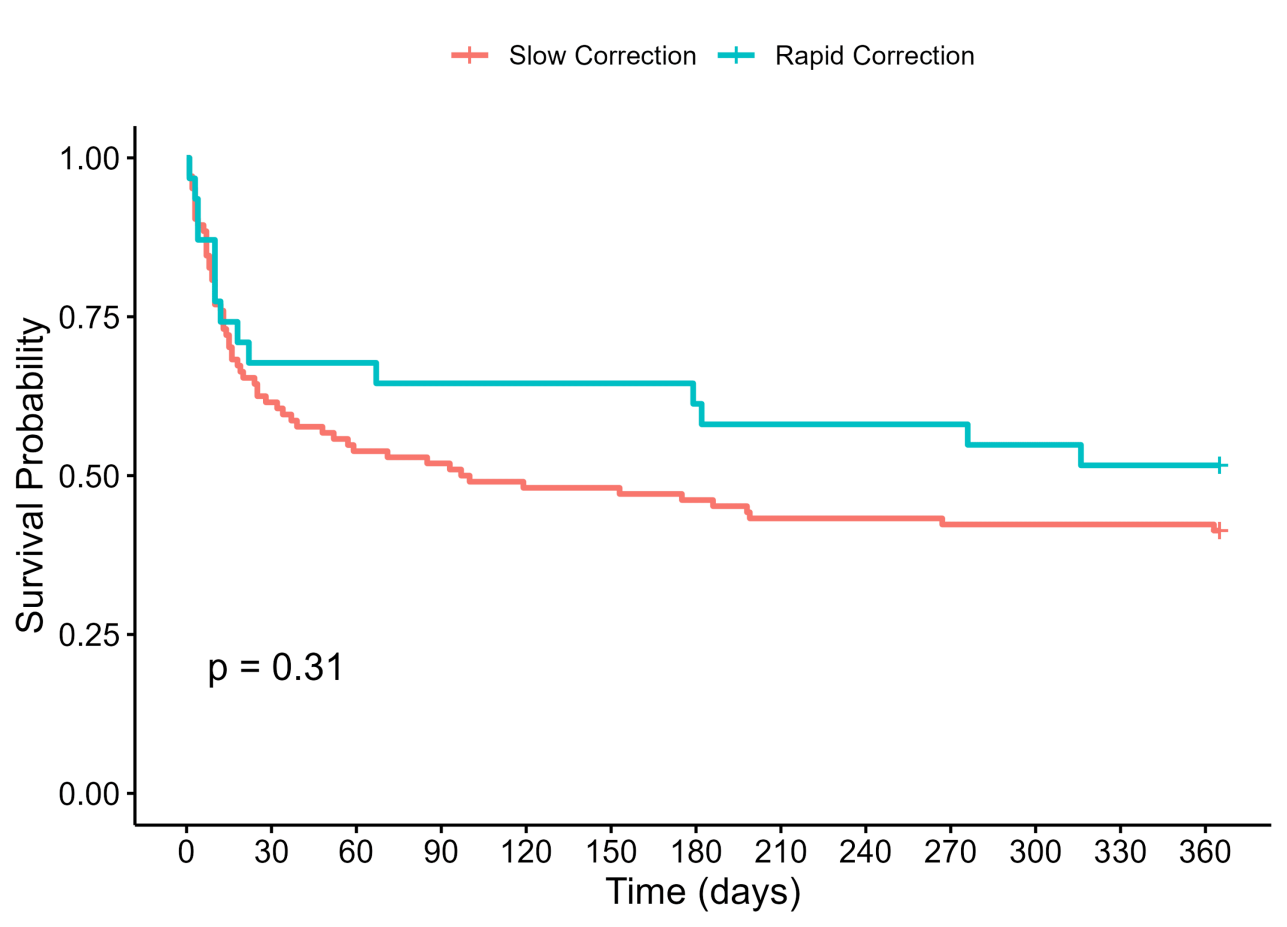
One-year survival curves for rapid versus slow correction groups

The slow correction group had higher 90-day (43% vs 33%, p=0.42) and one-year mortality rates (63% vs 52%, p=0.23) compared to the rapid correction group, although this was not statistically significant. Kaplan-Meier curves comparing 90-day and one-year survival between the rapid and slow correction groups are shown in Figure 1 and Figure 2, respectively. There were no significant differences between the curves. Of patients in the slow correction group, 70% had a maximal correction rate that exceeded 8 mmol/L/day. Mortality rates by maximum correction rates are shown in Table 2. There was no significant difference in mortality rates between slow and rapid maximal correction rate groups. Sensitivity analyses using 30 days and 90 days as presumed dates of death for those with unknown dates of death were consistent with the original analysis, with no significant difference in mortality between the two groups (Table 3). Repeat analysis with rapid rate of correction defined as ≥12 mmol/L/day also did not show any significant differences in mortality (Table 4). Cox regression analysis did not show any significant association with rates of correction and mortality when adjusted for age, gender, existing comorbidities, code status, and peak sodium levels (Table 5).

**Table 2:** Mortality by maximum rates of correction

Mortality	Maximum correction rate, n (%)		p-value
	< 8 mmol/L/day (n = 35)	≥ 8 mmol/L/day (n = 115)	
7 days	8 (23)	12 (10)	0.09
30 days	16 (46)	34 (30)	0.08
90 days	19 (54)	42 (37)	0.06
1 year	25 (71)	66 (57)	0.14

**Table 3:** Mortality by overall rates of correction, with unknown dates of death presumed deceased at 30 days and 90 days

Mortality	Overall correction rate, n (%)		p-value
	< 8mmol/L/day	≥ 8mmol/L/day	
With unknown dates of death presumed at 30 days			
30 days	53 (45)	12 (36)	0.43
With unknown dates of death presumed at 90 days			
30 days	40 (34)	10 (30)	0.83
90 days	63 (54)	13 (39)	0.17

**Table 4:** Mortality by maximal rates of correction, with rapid correction defined as ≥12 mmol/L/day and unknown dates of death presumed deceased at 180 days

	Maximum correction rate, n (%)		
Mortality	< 12 mmol/L/day	≥ 12 mmol/L/day	p-value
30 days	45 (34)	5 (29)	0.79
90 days	55 (41)	6 (35)	0.79
1 year	82 (62)	9 (53)	0.60

**Table 5:** Cox Proportional Hazards model, censored at 90 days, excluding patients with unknown dates of death

Variable	Hazard Ratio (95% CI)	p-value
Age	1.01 (0.99 - 1.03)	0.43
Sex – male	1.36 (0.84 - 2.21)	0.21
Charlson Comorbidity Index	1.12 (0.99 - 1.27)	0.06
Full code status on admission	1.21 (0.67 - 2.17)	0.53
Peak sodium levels	1.01 (0.96 - 1.06)	0.76
Rate of correction	1 (1.00 – 1.00)	0.27

Sodium correction rate and adverse events

Electronic medical records for the 33 patients with rapid correction were manually reviewed, with each patient being reviewed by two authors independently. One patient required a third author to act as a tiebreaker. No patients had documentation of adverse events attributed to the rapid rate of correction by the treating physicians at the time of admission. Two patients had iatrogenic hypernatremia from infusion of hypertonic saline in the setting of intracranial injury. No patients in the rapid correction group were identified as having worsening mental status, seizures, or imaging findings of cerebral edema (if performed), which could be attributed to the rate of correction on retrospective chart review by the authors.

## Discussion

Traditionally, the maximum rate of correction for hypernatremia has been considered the reverse of hyponatremia, with reviews and subspecialty training guides still advising a limit of 10-12 mmol/L per day [1,4]. This primarily stems from case reports of adverse outcomes with rapid correction of hypernatremia in neonates [13]. 

More recent research has indicated that this is unlikely to be applicable to the adult population. Using the Medical Information Mart for Intensive Care-III (MIMIC-III) dataset, Chauhan et al. examined critically ill patients in a tertiary center and found no association between the rate of correction and mortality [9]. Similarly, Feigin et al. reviewed over 4000 patients and again found no association with adverse outcomes with rapid correction of hypernatremia defined as exceeding 0.5 mmol/L/hour [8]. Indeed, their work supports earlier studies that suggested increased mortality with undercorrection of hypernatremia [14,15].

Our study joins this growing body of research that refutes the notion that rapid correction of hypernatremia causes harm. A recent meta-analysis performed agreed with this finding, although only six studies with severe hypernatremia greater than 155 mmol/L were included [16]. Conversely, it is becoming increasingly apparent that undercorrection of hypernatremia is associated with poor patient outcomes [14]. A direct consequence of undercorrection is increased length of stay (LOS), which is independently associated with increased mortality, in-hospital complications, and readmission rates [17]. Furthermore, severe hypernatremia alone can lead to encephalopathy and osmotic demyelination independent of correction rates [18]. Despite these findings, clinicians remain hesitant to aggressively correct hypernatremia, as confirmed in our study.

In our study, patient co-morbidities seem to play a significant role in the rate of correction in hypernatremia, as suggested by the higher CCI scores and the higher number of medications being taken in the slow correction group. For such patients, clinicians may elect to be more cautious with therapies to avoid exacerbating underlying conditions, such as heart failure. This cautious approach may inadvertently lead to similar treatment for patients without such comorbidities and lead to worse outcomes. Regardless of treatment, mortality rates were alarmingly high in both groups, with more than half of the patients deceased by the one-year mark. Interestingly, the proportions of patients who transitioned to comfort-oriented care were similar in both groups. This finding aligns with previously reported data, which suggests that hypernatremia is an independent marker of poor prognosis [19].

Strengths and limitations of the study

Our study aims to add to this growing body of evidence, with some key strengths compared to the larger studies published to date. Firstly, there is significant variation between studies published on what qualifies as rapid correction, with definitions ranging from 6 mmol/L/day to 12 mmol/L/day [20]. By adopting a more liberal cut-off of 8 mmol/L/day, our study was designed to be more sensitive to detect adverse events. Even with the more liberal definition of rapid correction, we were not able to identify any adverse neurological events attributable to the rapid rate of hypernatremia correction. Another strength of our study is the inclusion of smaller non-tertiary centers and long-term care facilities. Interestingly, despite five skilled nursing facilities and an extensive outpatient patient population, we were able to identify only six non-inpatient cases of severe hypernatremia. The inclusion of patients transitioning to comfort-oriented care (also known as “end-of-life care” or “hospice care”) is another unique strength of our study. About 40% of patients ultimately transitioned to comfort-oriented care overall, suggestive of poor underlying prognosis in patients with severe hypernatremia. 

Our study is limited primarily by the small sample size and the retrospective design. However, this is not unique to our study, as the overall incidence rates of severe hypernatremia are reported to be less than 0.5% [21]. Another limitation lies in the scope of data collection. Due to constraints in our electronic health records system, data extraction was manual, limiting our ability to capture key variables such as ICU admission status, specific treatments for hypernatremia, and comprehensive baseline laboratory values. These are all factors that are likely to affect outcomes but were not examined in our study. However, existing literature has not shown consistent or strong associations between these factors and patient outcomes [8,9]. Further research should focus on examining these confounding factors, particularly as artificial intelligence and advanced big data analytics become increasingly integrated into commercially available electronic medical records. Additionally, future studies could explore specific therapies for hypernatremia and their impact on patient outcomes.

## Conclusions

Our study suggests that rapid correction of hypernatremia, even above 8 mmol/L/day, is not associated with increased mortality or neurological complications. These findings challenge traditional correction rate limits of 8-12 mmol/L/day and provide further support for rapid correction in severe cases of hypernatremia. Guidelines should be updated to reflect the above, while future research should focus on identifying patient subgroups that may benefit from personalized approaches.
